# Flexible selection of diversified Na^+^/K^+^-ATPase α-subunit isoforms for osmoregulation in teleosts

**DOI:** 10.1186/s40851-016-0050-7

**Published:** 2016-08-03

**Authors:** Marty Kwok-Shing Wong, Supriya Pipil, Haruka Ozaki, Yutaka Suzuki, Wataru Iwasaki, Yoshio Takei

**Affiliations:** 1 Atmosphere and Ocean Research Institute, The University of Tokyo, Kashiwa, Chiba Japan; 2 Department of Biological Sciences, Graduate School of Science, The University of Tokyo, Tokyo, Japan; 3 Department of Computational Biology, Graduate School of Frontier Sciences, The University of Tokyo, Tokyo, Japan; 4 Bioinformatics Research Unit, Advanced Center for Computing and Communication, RIKEN, Wako, Saitama Japan

**Keywords:** Na^+^/K^+^-ATPase, Nomenclature, Evolution, Isoforms, Osmoregulation, Teleost, Gene duplication, Transcription-induced chimerism

## Abstract

**Background and methods:**

Multiple Na+/K+-ATPase (NKA) α-subunit isoforms express differentially in response to salinity transfer in teleosts but we observed that the isoform nomenclature is inconsistent with the phylogenetic relationship of NKA α-genes. We cloned the catalytic NKA α-subunit isoforms in eels and medaka, analyzed the time course of their expressions in osmoregulatory tissues after transfer from freshwater (FW) to seawater (SW), and performed phylogenetic analyses to deduce an evolutionary scenario that illustrates how various duplication events have led to the current genomic arrangement of NKA α-genes in teleosts.

**Results and discussion:**

Five and six α-subunits were cloned in eels and medaka respectively. In eels, the commonly-reported α1a and α1b isoforms were absent while the α1c isoform was diversified instead (α1c-1, α1c-2, α1c-3, α2, and α3 in eels). Phylogenetic estimation indicated that the α1a and α1b isoforms from salmon, tilapia, and medaka were generated by independent duplication events and thus they are paralogous isoforms. Re-examination of expression changes of known isoforms after salinity challenge revealed that the isoforms selected as predominant SW-types varied among teleost lineages. Diversification of α1 isoforms occurred by various types of gene duplication, or by alternative transcription among tandem genes to form chimeric transcripts, but there is no trend for more α1 copies in euryhaline species. Our data suggest that the isoform switching between FW (α1a predominates) and SW (α1b predominates) that occurs in salmonids is not universal in teleosts. Instead, in eels, α1c-1 was the major α-subunit upregulated gill, intestine, and kidney in SW. Localization of both NKA mRNA and protein showed consistent upregulation in gill and intestine in SW eels, but not in renal distal and collecting tubules, where low transcript expression levels were accompanied by high protein levels, suggesting a tissue-specific translational regulation that determines and fine-tunes the NKA expression. In medaka, α1b was upregulated in SW in anterior intestine while most other α-subunit isoforms were less responsive to salinity changes.

**Conclusion:**

By integrating gene expression and phylogenetic results, we propose that the major NKA α-subunits for SW acclimation were not ancestrally selected, but rather were flexibly determined in lineage-specific fashion in teleosts.

## Background

Na^+^/K^+^-ATPase (NKA) is the key enzyme involved in generating the driving force for Na-coupled ion transport in most animal systems. It also plays an important role in maintaining the resting membrane potential, which consumes 2/3 of the total energy used by neurons [1]. Located mostly on the basolateral membrane, the NKA pumps out 3 Na^+^ and in 2 K^+^ at the expense of ATP hydrolysis, and the enzyme is usually enriched in active ion transporting epithelia in the gill, intestine, and kidney of fishes [2]. NKA is composed of two major subunits: the α-subunit is the catalytic unit and the β-subunit is crucial for the structural and functional maturation of NKA and modulates its transport properties [3]. As the α-subunits are the major functional machineries for the NKA proteins, their expressions are highly correlated to transporting functions of some osmoregulatory epithelia such as gill and intestine in fishes.

NKA is the most extensively studied transporter in teleost osmoregulation, and several α-subunit isoforms have been identified. Among these isoforms, it is well-known that the salmonid NKA α1a and α1b isoforms are expressed reciprocally in freshwater (FW) and seawater (SW) ionocytes [4–6]. NKA α1a is highly expressed in FW and downregulated in SW while NKA α1b is low in FW and upregulated in SW, whereas NKA α1c is unaffected by salinity transfer [4]. Similar switching of NKA α1a and α1b isoforms has also been reported in tilapia based on partial sequences including an isoform-specific substitution on the fifth transmembrane helix [7]. However, the identity of α1a and α1b was not thoroughly studied, as tilapia possesses more α1a isoforms than salmonids and whole protein sequences were not characterized previously. A comprehensive study of salmonid NKA α-subunit evolution showed that tilapia α1a and α1b are not orthologous to those of salmonids; however, strong selection pressure could have led to some degree of parallel evolution among α1-isoforms [8]. Furthermore, it was recently reported that Japanese medaka lack the α1a and α1b switching pattern and that only α1b was upregulated in the gill of SW individuals [9]. The phylogeny of medaka NKA α1a and α1b, similar to the case in tilapia, did not support an orthologous relationship among different species. As more genome and expression data are published, we observed alternative selection of α-subunits by teleosts. Thus the α1a and α1b switching scenario should be revisited.

Recent expansion of next-generation sequencing (NGS) technology has fueled many breakthroughs via genomic and transcriptomic analyses. Among these methods, RNA-seq is powerful for the discovery of functional important genes by analysis of differential expressed genes (DEGs) in cells and tissues under different treatments. Many studies have attempted to analyze the DEGs in the tissue of salinity-acclimated fish to understand the molecular basis of osmoregulation [10–15], but NKA α-subunit isoforms were rarely described in these studies, as the short sequences from Illumina sequencing are indiscriminately matched to the closely resembled isoforms, thus limiting the accuracy of RNA-seq quantification. We combined cloning and genome information of eel and medaka to analyze the phylogenetic relationship as well as the time course of expression levels of NKA α-subunit isoforms in various tissues after salinity transfer. These time-course data are valuable, as most other studies focused only on salinity-acclimated fishes while some of the changes in the expression of NKA α-subunit isoforms could be transient. Along with other known sequences and expression profiles in different salinities from literature, we aim to establish an evolutionary scheme for the emergence of teleost NKA α-subunits by a combined perspective of phylogeny, genomics, and physiological functions in various teleost representatives in order to clarify the nomenclature of these isoforms.

## Methods

### Animal husbandry and sampling

Juvenile Japanese eel (*Anguilla japonica*, 170 - 210 g) were obtained from a local eel farm and kept in a recirculating aquarium system in the Atmosphere and Ocean Research Institute, The University of Tokyo. The water was maintained at 18 °C and the eel was exposed to a 14 h:10 h light/dark cycle throughout the experiment. FW was 0 ‰ with Na^+^ (1.07 mM), Ca^2+^ (0.48 mM), and Mg^2+^ (0.27 mM) determined by an atomic absorption spectrometer (Hitachi 180-80, Japan) while Cl^-^ was too low to be measured by a chloride meter (Labconco 4425000, Missouri). Natural SW was obtained from the Kuroshio Current at Hachijō-jima and the salinity was 35 ‰ with Na^+^ (432 mM), Cl^-^ (558 mM), Ca^2+^ (9.3 mM), and Mg^2+^ (63.1 mM). Measured osmolality values of FW and SW were 0 and 1019 mOsm respectively by a vapor osmometer (VAPRO 5520, ELITech, Puteaux). The eels were not fed during the entire course of experiment. Using a fish net, FW eels were transferred to FW (control-transfer) or SW (treatment-transfer) and various tissues including gill, esophagus, stomach, anterior intestine, middle intestine, posterior intestine, rectum, and trunk kidney were obtained 3 h, 12 h, 1d, 3d, and 7d after the transfer (*n* = 5 each). Pre-transfer eels were taken as FW intact control (*n* = 5).

Japanese medaka (*Oryzias latipes*, HdrR strain) were bred and kept in a recirculating aquarium system maintained at 25 °C with 14 h:10 h light/dark cycle. Medaka were fed daily with freshly hatched brine shrimp. The sources and compositions of FW and SW in medaka experiment were the same as those of eel. FW medaka were transferred to FW (control-transfer) or 50 % SW (treatment-transfer) for 1 h, 3 h, 1d, and 7d. Pre-transfer medaka were taken as FW intact control (*N* = 6 in each group). During the samplings, eel and medaka were anesthetized in 0.1 % MS-222 (ethyl 3-aminobenzoate methanesulfonate, Sigma-Aldrich Chemicals, St Louis, MO, USA) neutralized with NaHCO_3_. Fish were subsequently sacrificed by decapitation and required tissues were swiftly removed and snap-frozen in liquid nitrogen. Tissues were stored at –80 °C until further use. All animal studies were performed according to the Guideline for Care and Use of Animals approved by the Animal Experiment Committee of the University of Tokyo.

### Transcriptome analysis by RNA-seq

The total RNA of medaka and eel were extracted using Isogen (Wako Pure Chemical Industries, Osaka, Japan), reverse transcribed to cDNA libraries using TruSeq RNA Sample Preparation v2 (Illumina Inc, CA), and sequenced by Illumina HiSeq 2500 (101 bp, paired-end) in the Laboratory of Functional Genomics, The University of Tokyo, according to the manufacturer’s protocols. RNA-seq of the gill and intestine samples from medaka was performed as outlined previously [15]. For eel RNA-seq, the sequenced reads were mapped to the Japanese eel genome [16] using TopHat (version 2.0.9) [17]. The mapped reads were pooled for each condition, and genome-guided transcriptome assembly was performed to reconstruct the eel transcripts using Cuffilink version 2.1.1. The assembled transcripts were merged using Cuffmerge, and the merged transcripts were used for quantifying gene expression levels.

For each eel transcript, open reading frames (ORFs) were predicted using EMBOSS getorf (version 6.6.0) [18] with the parameter “-minsize 300”. Then, for each gene, the longest ORF among ORFs predicted from all transcripts belonging to the gene was selected, and the translated amino acid sequence of the ORF was used for the following blast search. The reciprocal blast search was performed using amino acid sequences of medaka and eel using BLASTP in NCBI-BLAST+ (version 2.2.29+) [19] with the parameter “-evalue 1e-5”. Longest amino acid sequence for each medaka gene in Ensembl annotation (release 74) was used. Reciprocal BLAST best hits (RBBH) in terms of E-value were defined as RBBHs between medaka and eel. Gene annotation of eel was guided using the medaka genome as reference database with RBBH. Only genes with at least 10 reads in at least two samples were used in the following analysis, and low-count genes were removed. The relative gene expression was normalized using the iDEGES method implemented in the TCC package (version 1.2.0) [20]. Transcriptome of intestine and gill in medaka and eel were deposited in DDBJ database with accession number DRA004257 and DRA004258 respectively.

### NKA isoform cloning and phylogenetic analysis

Initially, we searched for putative NKA sequences in the draft genome of Japanese eel using BLASTn, and obtained independent scaffolds (scaffold 12167, 20013, 2250, 15826, 2700, and 8515) and transcript data (t20531, t13768, t17728, and t10968) [16]. Total RNA was extracted from the frozen tissues using Isogen, treated with DNase I (Life Technologies, Grand Island, NY, USA) to remove genomic DNA, and subsequently reverse transcribed into cDNA by iScript cDNA Synthesis Kit (Bio-Rad Laboratories, Inc, Hercules, CA, USA) according to the manufacturer’s protocols. To obtain full-length cDNA sequences, specific primers were designed on various regions of the predicted sequences and 3′-RACE was performed to obtain the unknown sequences and 3′-untranslated regions (UTRs). 3′-RACE cDNA templates were prepared from FW and SW eel gills using the SMART cDNA Cloning kit. Long distance PCR was performed to amplify the 3′-ends of NKA isoforms using a KOD plus reagent kit (Toyobo, Osaka Japan) according to the high GC reaction profiles of the manufacturer’s protocol. All sequencing reactions were performed using BigDye Terminator 3.1 cycle sequencing kit (ThermoFisher Scientific, Waltham, MA, USA) according to the company protocols.

The deduced protein sequences of representative vertebrate NKA α-subunits were collected from cloning and BLAST search from Ensembl release 84 [21] and NCBI nucleotide databases [22] and were used to reconstruct the phylogenetic relationship. The sequences were aligned using MUSCLE with default settings in MEGA version 6, and the best protein model was searched and subsequently used in the phylogenetic analysis. Phylogenetic trees were constructed using the maximum likelihood method in MEGA version 6 based on the LG model [23]. A discrete Gamma distribution was used to model evolutionary rate differences among sites (5 categories (+G, parameter = 0.3816)). The rate variation model allowed for some sites to be evolutionarily invariable ([+I], 3.5494 % sites). Bootstrap tests were performed with 1000 replicates to verify the robustness of the phylogenetic relationships.

Synteny analysis was performed among the neighbor orthologous genes of *atp1a1* (α1), *atp1a2* (α2), and *atp1a3* (α3) among Japanese eel, medaka, tilapia, and zebrafish to reveal the genomic organization of different isoforms generated by independent and/or genome duplications.

### Quantification of NKA isoform expressions during time-course SW transfer

After obtaining the nucleotide sequence information, we designed specific primers for quantitation of the isoform expressions according to the mismatch found in the alignment among different isoforms, especially in the 3′-untranslated region of eel isoforms. Reactions were carried out in 10 μL scale using Kappa SYBR 2X PCR mix (KAPA Biosystems, Wilmington, Del, USA) and ABI 7900HT Fast Real Time PCR System (Life Technologies, CA, USA). The amplification of a single amplicon was confirmed by analyzing the melting curve after cycling. Elongation factor 1 alpha (*eef1a*) was used as an internal control to normalize the gene expressions among different samples. Relative gene expression of target genes was quantified by the 2–[delta] [delta] Ct method where [delta] [delta] Ct = [delta] Ct,target - [delta] Ct,*eef1a*. Primer sequences for quantitative PCR are listed in Table 1. The eel and medaka NKA α-subunit isoforms were quantified comprehensively in different tissues under a SW time-course transfer scheme. Time-matched controlled transfer (FW-FW) data were also included to remove any handling stress-related artefacts.

**Table 1:** Primer sequences for real time PCR

GenBank/Ensembl accession number	Gene & Primer names	Forward (F) Reverse (R)	Oligo sequences (5′ to 3′)
Japanese eel			
KU976438	*atp1a1c*-1 (α1c-1)	F	CTGTATGATGAAGCCCGAAGAT
		R	AATGTGTGATGCAGGCAGTA
KU976439	*atp1a1c*-2 (α1c-2)	F	GTACTACTGAAGCTTCCTGTCTG
		R	CACATCCAGAAGTTACTGAGGTTA
KU976440	*atp1a1c*-3 (α1c-3)	F	CCCTATTCACTCCTCATCTTCATT
		R	GCACTAGTAGTACGTCTCTCTCT
KU976441	*atp1a2* (α2)	F	GACTCGACGCAACTCTGTTT
		R	ACGAAGATCAGGAGGCTGTA
KU976442	*atp1a3* (α3)	F	CCTCATCTTTGTGTACGATGAGATA
		R	GATGGTGTGAAGCAGAAGAAATG
AB593812	*eef1a* (ef1α)	F	CTGAAGCCTGGTATGGTGGT
		R	ACGACGGATTTCCTTGACAG
Medaka			
ENSORLG00000018557	*atp1a1a*.4 (2 of 2) (α1a)	F	TAAATGAAAGGCTCATCAGTATGGC
		R	GTCTTCCAGGTCGTTTACAGG
ENSORLG00000002122	*atp1a1a*.4 (1 of 2) (α1b)	F	CTGATCTGCGAATTGTCTCCTC
		R	AGTCTGGGGTTCGGATTCAC
ENSORLG00000002047	*atp1a1b* (α1c)	F	CTGGCTGGATTCTTCACCTATT
		R	CCTCCAGGTCGTTGATGTATTT
ENSORLG00000002639	*atp1a2a* (α2)	F	TGCTGTGGGTAAATGCCGCTCT
		R	TTGGCTGTGATTGGGTGG
ENSORLG00000007036	*atp1a3a* (α3a)	F	GCTTATGAAGCAGCAGAAAGTG
		R	CCTATTTGTCCGTAGGCGATAC
ENSORLG00000013191	*atp1a3b* (α3b)	F	TGAGGAAATATGCAGGAAGTTGAAT
		R	CAGAAACTCTGCTGCCTTTGC
ENSORLG00000007614	*eef1a* (ef1α)	F	AGATGCACCACGAGTCTTTAC
		R	GACGTATCCACGACGGATTT

### Immunohistochemistry and in situ hybridization NKA in eel gill, intestine, and kidney

The gill, intestine, and kidney of FW and SW eels were fixed in 4 % paraformaldehyde in 0.1 M phosphate buffer at pH 7.4 for 1 day at 4 °C. Tissues were dehydrated through serial alcohol/xylene solutions and subsequently embedded in Paraplast (Leica Microsystems, Wetzlar, Germany). Paraffin sections (5 μm) were made and mounted on MAS-coated slides (Matsunami, Osaka, Japan) and stored at 4 °C until use. For IHC, tissue sections were deparaffinized and rehydrated to deionized water by serial xylene/alcohol series. The sections were treated with 0.2 % H_2_O_2_ in methanol for 30 min to inactivate endogenous peroxidase activity and then non-specific sites were blocked by 2 % normal goat serum in PBS (pH7.4) for 60 min. Monoclonal NKA antibody (a5, DSHB, IA, USA) was diluted (1:10,000) in PBS containing 2 % normal goat serum and 0.01 % NaN_3_ and incubated with the sections at 4 °C for 16–18 h in a moist chamber saturated with water vapor. Immunoreactive signals were developed using a Vectastain ABC Elite kit (Vector Laboratories, CA, USA) and 3,3′-diaminobenzidine as the color reagent according to manufacturer’s protocols. Sections were counterstained with hematoxylin after color development.

For ISH, sections were deparaffinized in xylene and rehydrated by serial alcohol solutions. They were treated with proteinase K (5 μg/mL) for 10 min and then post-fixed in 4 % paraformaldehyde in 0.1 M phosphate buffer at pH 7.4. The sections were equilibrated in hybridization buffer (5x SSC, 50 % formamide) at 58 °C for 2 h. RNA probes were designed at the 3’-end of the mRNA, utilizing most of the UTRs to increase specificities. Sense and antisense probes for eel NKA α-subunit isoforms were prepared using a Digoxigenin RNA Labeling Kit (Roche Applied Science, IN, USA) according to the manufacturer’s protocol. The RNA probes (50 ng/slide) were diluted in hybridization buffer supplied with calf thymus DNA (40 μg/mL) and denatured at 85 °C for 10 min. Denatured RNA probes were spread on the sections and the slides were incubated at 58 °C for 40 h in a moist chamber saturated with hybridization buffer. Probe signals were developed using a Digoxigenin Nucleic Acid Detection Kit (Roche Applied Science, IN, USA) according to the manufacturer’s protocol.

### Statistical analysis

For quantitative PCR, the time-course of gene expressions in different tissues of FW and SW eels and medaka were analyzed by two-way ANOVA followed by Tukey’s multiple comparisons and salinity groups with *P* < 0.05 were considered as significantly different (GraphPad Prism Ver. 6 for Windows, San Diego, CA, USA).

## Results

### Sequence characteristics and phylogeny of NKA isoforms in teleosts

**Fig. 1:**
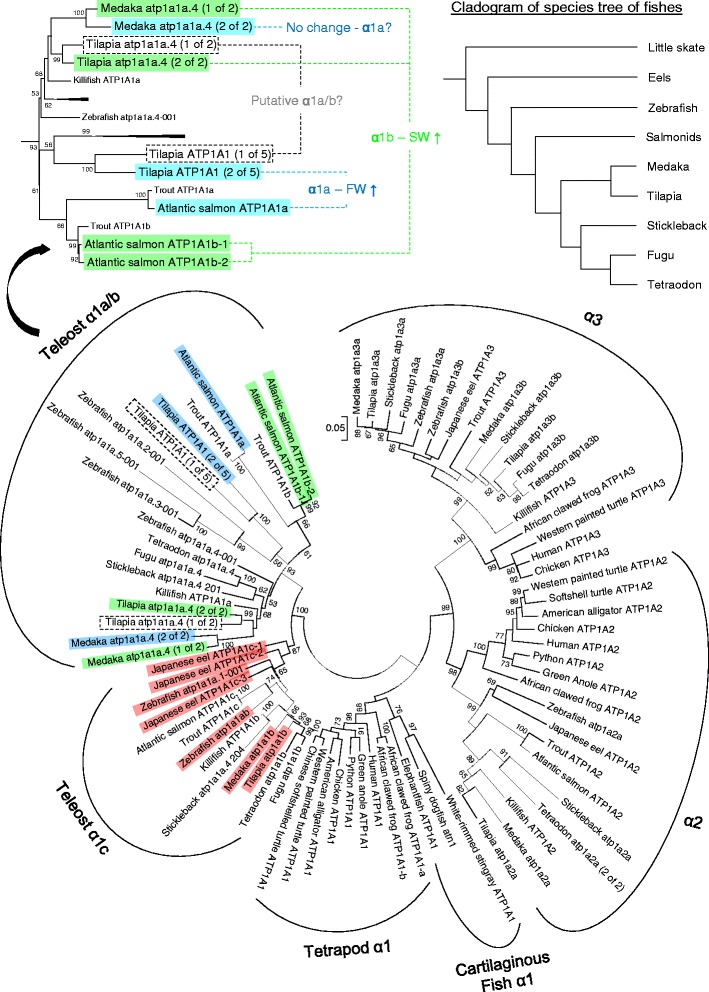
Phylogenetic tree of representative vertebrate NKA α-subunits depicted by maximum likelihood method. The deduced proteins of NKA α-subunits from prediction and cloned sequences are included in the analysis. Blue, green, and red highlights indicate the α1a, α1b, α1c isoforms respectively in Atlantic salmon, tilapia, medaka, and eel with quantitative real time PCR data from literatures. Dashed boxes indicate putative α1a/b that have not been characterized in tilapia. The top left panel shows the enlarged α1a/b clade in teleost to indicate the paraphyletic relationship among known α1a and α1b in known species. The top right panel shows a cladogram of contemporary species tree that indicates the phylogenetic relationship of the teleost representative used in this study (summarized from [24]). Each taxon is named with genome- or GenBank-annotated identity. Numbers on the branches are the bootstrap values of 1000 replicates. Scale bar represents 5 % amino acid substitution

We collected most available NKA α-subunits sequences via cloning and sequence search for phylogenetic analysis to elucidate their evolutionary relationships. The resulting phylogenetic tree showed the representative vertebrate NKA α-subunits to be clustered into distinct clades including teleost α1, tetrapod/cartilaginous fish α1, α2, and α3 (Fig. 1). In the teleost α1 clade, two subclades could be clearly identified, and according to the nomenclature of salmonid NKA α1 isoforms [5], we tagged these two major subclades as teleost α1a/b and α1c. We cloned three α1, one α2, and one α3 in eel. The phylogeny results indicated that all eel α1 isoforms belong to α1c subclade. We therefore named them α1c-1, α1c-2, and α1c-3. We also analyzed the medaka NKA α-subunit isoforms and found two α1a/b, one α1c, one α2 and two α3 isoforms (Fig. 1; Table 2 for accession numbers). Tetrapod α1 and cartilaginous fish α1 isoforms were highly conserved, suggesting that the tetrapod α1 could have retained the ancestral characteristics while the teleost α1 diverged and underwent lineage-specific radiation. The α2 and α3 isoforms clustered into distinct clades and most teleosts possess a single α2 and two α3 (α3a and α3b). It is known that tetrapods possess NKA α4 (*atp1a4*), but this was a lineage-specific duplication from α2. We therefore did not include the sequences in this analysis. The teleost α1 subclade is enlarged in Fig. 1 to indicate the non-monophyletic relationship among different salinity-sensitive α1a and α1b in salmon, tilapia, and medaka. A simplified cladogram of the species tree of fishes is shown to indicate the contemporary phylogenetic classification of fish species used in the present study for the comparison between gene and species diversification [24].

**Table 2:** List of accession numbers of NKA α-subunits used in phylogenetic analysis. Known isoforms are matched with accession numbers and annotation. Types of isoform duplication are summarized

Species	Annotation	Accession Number	Isoform description	Duplication types
Human	ATP1A1	NM_000701		
	ATP1A2	NM_000702		
	ATP1A3	NM_152296		
Chicken	ATP1A1	NM_205521		
	ATP1A2	NM_205476		
	ATP1A3	NM_205475		
American alligator	ATP1A1	XM_006261470		
	ATP1A2	AKHW02002543		
Green anole	ATP1A1	XM_008119744		
	ATP1A2	XM_008124767		
	ATP1A3	ENSACAG00000015035		
Python	ATP1A1	XM_007435293		
	ATP1A2	XM_007429082		
	ATP1A3	XM_007437572		
Chinese soft-shelled turtle	ATP1A1	XM_006132885		
	ATP1A2	XP_014425124		
Western painted turtle	ATP1A1	XM_005292679		
	ATP1A2	XM_005293763		
	ATP1A3	XM_005312502		
African clawed frog	ATP1A1-a	NM_001090595		
	ATP1A1-b	NM_001089111		
	ATP1A2	NM_001089643		
	ATP1A3	NM_001086971		
Elephantfish	ATP1A1	XM_007891602		
White-rimmed stingray	ATP1A1	KF724944		
Spiny dogfish	atn1	AJ781093		
Zebrafish	atp1a1a.1	ENSDARG00000002791		Merged α1 gene
	atp1a1a.1-001 (transcript)	ENSDART00000081047	α1a/b-1	Tandem duplication from ancestral α1a/b
	atp1a1a.1-201 (transcript)	ENSDART00000161781	α1c-1 + α1a/b-1 + α1a/b-4 (chimera)	Chimeric transcript among atp1a1a.4-001, atp1a1a.1-001, & atp1a1a.5-001
	atp1a1a.2	ENSDARG00000007739		Merged α1 gene
	atp1a1a.2-001 (transcript)	ENSDART00000006449	α1a/b-2	Tandem duplication from ancestral α1a/b
	atp1a1a.2-201 (transcript)	ENSDART00000166968	α1a/b-1 + α1a/b-2 + α1a/b-3 (chimera)	Chimeric transcript among atp1a1a.1-001, atp1a1a.2-001, & atp1a1a.3-001
	atp1a1a.3	ENSDARG00000039131		
	atp1a1a.3-001 (transcript)	ENSDART00000103850	α1a/b-3	Tandem duplication from ancestral α1a/b
	atp1a1a.4	ENSDARG00000001870		
	atp1a1a.4-001 (transcript)	ENSDART00000048828	α1c-1	Independent duplication
	atp1a1a.5	ENSDARG00000040252		
	atp1a1a.5-001 (transcript)	ENSDART00000006013	α1a/b-4	Tandem duplication from ancestral α1a/b
	atp1a1b	ENSDARG00000019856	α1c-2	Independent duplication
	atp1a2a	ENSDARG00000010472	α2	
	atp1a3a	ENSDARG00000018259	α3a	3RWGD
	atp1a3b	ENSDARG00000104139	α3b	3RWGD
Tilapia	atp1a1 (1 of 5)	ENSONIG00000012358	α1a-1	Tandem duplication from ancestral α1a
	atp1a1 (2 of 5)	ENSONIG00000012375	α1a-2	Tandem duplication from ancestral α1a
	atp1a1a.4 (1 of 2)	ENSONIG00000012396	α1b-2	Tandem duplication from ancestral α1b
	atp1a1a.4 (2 of 2)	ENSONIG00000012431	α1b-1	Tandem duplication from ancestral α1b
	atp1a1b	ENSONIG00000012456	α1c	
	atp1a2a	ENSONIG00000015107	α2	
	atp1a3a	ENSONIG00000004959	α3a	3RWGD
	atp1a3b	ENSONIG00000013081	α3b	3RWGD
Stickleback	atp1a1a.4	ENSGACG00000014324		Merged α1 gene
	atp1a1a.4-201 (transcript)	ENSGACT00000018945	α1a/b	Ancestral α1a/b
	atp1a1a.4-202 (transcript)	ENSGACT00000018949	α1a/b + α1c (chimera)	Chimeric transcript between ancestral α1a/b and α1c
	atp1a1a.4-203 (transcript)	ENSGACT00000018954	α1a/b	Alternative transcript of ancestral α1a/b
	atp1a1a.4-204 (transcript)	ENSGACT00000018961	α1c	Ancestral α1c
	atp1a2a	ENSGACG00000017683	α2	
	atp1a3a	ENSGACG00000001959	α3a	3RWGD
	atp1a3b	ENSGACG00000009524	α3b	3RWGD
Medaka	atp1a1a.4 (2 of 2)	ENSORLG00000018557	α1a	Independent duplication from atp1a1a.4 (1 of 2)
	atp1a1a.4 (1 of 2)	ENSORLG00000002122	α1b	
	atp1a1b	ENSORLG00000002047	α1c	
	atp1a2a	ENSORLG00000002639	α2	
	atp1a3a	ENSORLG00000007036	α3a	3RWGD
	atp1a3b	ENSORLG00000013191	α3b	3RWGD
Killifish	atp1a1a	AY057072	α1a	
	atp1a1b	AY430089	α1b	
	atp1a2	AY057073	α2	
	atp1a3	XM_012855590	α3	
Fugu	atp1a1a.4	ENSTRUG00000013282	α1a/b	
	atp1a1b	ENSTRUG00000012850	α1c	
	atp1a3b	ENSTRUG00000008243	α3a	
	atp1a3a	ENSTRUG00000002904	α3b	
Tetraodon	atp1a1a.4	ENSTNIG00000006257	α1a/b	
	atp1a1b	ENSTNIG00000006396	α1c	
	atp1a2a (2 of 2)	ENSTNIG00000004890	α2	
	atp1a3b	ENSTNIG00000008007	α3	
Rainbow trout	atp1a1a	AY319391	α1a	
	atp1a1b	AY319390	α1b	
	atp1a1c	AY319389	α1c	
	atp1a2	AY319387	α2	
	atp1a3	AY319388	α3	
Atlantic salmon	atp1a1a	AIB08901	α1a	
	atp1a1b-1	AIG14471	α1b	Salmon specific tetraploidy
	atp1a1b-2	AIG14472	α1b	Salmon specific tetraploidy
	atp1a1c	AIB08902	α1c	
	atp1a2	AY692147	α2	
Japanese eel	atp1a1c-1	KU976438	α1c-1	Independent duplication from ancestral α1c
	atp1a1c-2	KU976439	α1c-2	Independent duplication from ancestral α1c
	atp1a1c-3	KU976440	α1c-3	Independent duplication from ancestral α1c
	atp1a2	KU976441	α2	
	atp1a3	KU976442	α3	

### Synteny analysis of NKA isoforms in teleosts

**Fig. 2:**
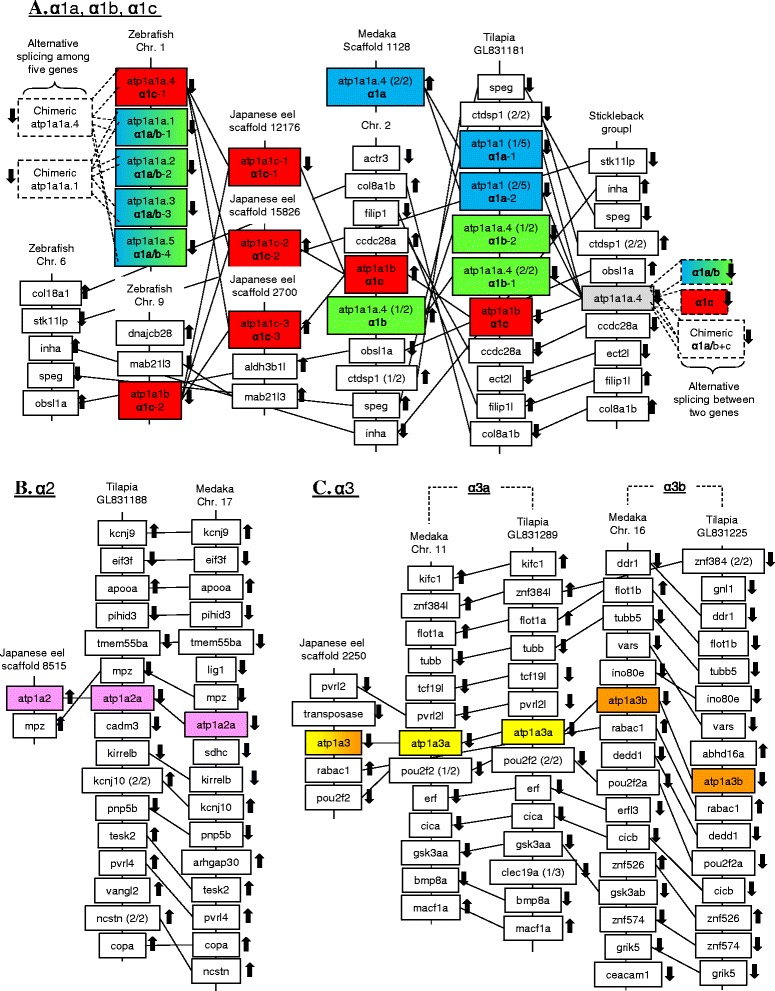
Synteny of the genomic regions of (**a**) α1- *atp1a1*, (**b**) α2- *atp1a2*, and (**c**) α3- *atp1a3*. NKA gene isoforms among various species were shaded. Various *atp1a1* isoforms in tilapia are located on a side by side locus on same chromosomes, suggesting their formation were the results of tandem duplications. Stickleback α1 isoforms was hidden at the genomic level as alternative transcription between α1a/b and α1c generates a chimeric α1 isoform. Five zebrafish α1 genes on Chromosome 1 are alternatively transcribed to form seven known α1 transcripts. Teleost α3a and α3b (*atp1a3a* and *atp1a3b*) are located on different chromosomes with highly conserved neighbor gene synteny in both tilapia and medaka, suggesting that the isoforms were the result of the 3R whole genome duplication in teleosts. Various colors indicate different isoforms and gradient colors indicate the isoforms cannot be distinguished: α1a (*blue*); α1b (*green*); α1a/b (gradient *blue* to *green*); α2 (*magenta*); α3a (*yellow*); α3b (*brown*); and α3a/b (gradient *yellow* to *brown*). The chromosomal directions of the genes are indicated by arrows. Connecting lines indicate orthologous genes

At the chromosomal level, the α1a/b and α1c are located side by side on the same chromosome and surrounded by similar set of neighbor genes in tilapia and medaka (Fig. 2a). The eel NKA α1c-1, α1c-2, and α1c-3 were identified on different scaffolds, and the scaffolds are mostly too short to reveal any syntenic relationship. However, one synteny gene (*mab21l3*) on eel α1c-3 scaffold was identified in zebrafish chromosome 9, which harbors α1c. Whether various eel α1c isoforms are located on the same or different chromosomes requires a more complete genome sequencing and assembly. The European eel genome was also searched, but the corresponding scaffolds are shorter than those of Japanese eel. We thus focused only on Japanese eel genomic contigs. Medaka α1a and α1b are located on different scaffolds but α1c is located next to α1b. Although α1a and α1b were previously described in tilapia, the genomic sequence showed that two additional isoforms of α1a and α1b are present, along with a single α1c. We named these previously identified isoforms α1a-1 and α1b-1, and named the additional isoforms α1a-2 and α1b-2, respectively (Fig. 2a). All five tandem NKA α1 isoforms in tilapia are located immediately next to each other on the same scaffold, which are surrounded by neighbor genes sharing high syntenic relationships with those of medaka. In stickleback, only one α1 gene is annotated in the genome but it is actually composed of two adjacent α1 genes with the same orientation that merged by alternative transcription. Five α1 genes are tandemly located on chromosome 1 in zebrafish, with the same orientation, but phylogeny results did not support that the tandem duplication is homologous to those in tilapia. Furthermore, the syntenic relationship at the region of the tandem α1 genes on chromosome 1 in zebrafish appeared to be lost, as we located the syntenic region in chromosome 6 (Fig. 2a). The syntenic region on zebrafish chromosome 6 contains several genes homologous to the neighbor genes of α1 tandems in medaka, tilapia, and stickleback, but no α1 genes are present in this region.

The eel α2 genomic scaffold was short but one neighbor gene (*mpz*) is syntenic to those of medaka and tilapia (Fig. 2b). The neighbor gene position and orientation of α2 between medaka and tilapia are highly syntenic, and we cannot identify any duplication region in various teleost genomes so far, despite the 3R whole genome duplication event. The composition and organization of the genes vicinity of α3a and α3b were highly similar in the medaka and tilapia scaffolds (Fig. 2c). The synteny structure suggests chromosomal duplication, thus teleost α3 isoforms were likely a result of the teleost-specific 3R whole genome duplication. The eel α3 scaffold is relatively long and we identified several syntenic genes that can be found on medaka chromosomes harboring α3a and α3b. *pvrl2* and *pou2f2* are found along with α3a on medaka chromosome 11, while *rabac1* is found along with α3b on medaka chromosome 16. The eel α3 is clustered in the α3a subclade on the phylogenetic tree but the scaffold share some syntenic relationships to both chromosomes harboring α3a and α3b in medaka.

### Transcription-induced chimerism among NKA α1 isoforms

**Fig. 3:**
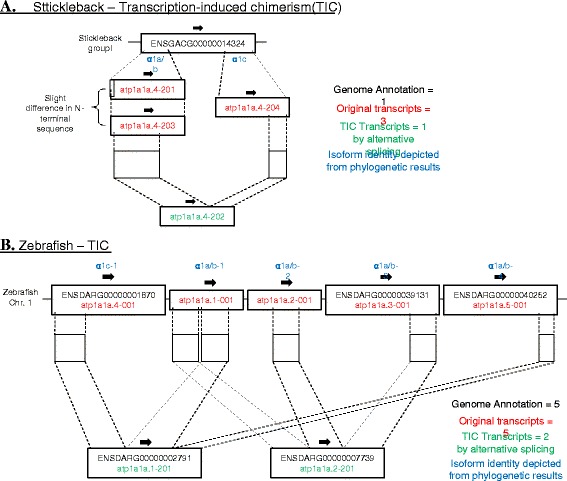
Schematic diagram showing the transcription-induced chimerism (TIC) at chromosomal regions of NKA α1 in (**a**) stickleback, and (**b**) zebrafish. In stickleback (**a**), two original α1 genes (*red*) were hidden in a single annotation of *atp1a1* as alternative transcripts. The chimeric transcript atp1a1a.4-202 (*green*) formed by alternative splicing across two tandem genes is the result of TIC. In zebrafish (**b**), five original α1 genes (*red*) were present on the chromosome but they were not corresponding to the five annotated genes in the genome. Instead, two TIC transcripts (*green*) were interpreted as genes while two original genes were interpreted as alternative transcripts. Various color displays on the right shows different definitions of the annotation, genes, and transcripts. Blue color indicates isoform identity depicted from phylogenetic results

As we found that the genome annotation for genes and transcripts were confusing in some species such as stickleback and zebrafish, we analyzed the detail composition of genes and transcripts in these two species (Fig. 3). Transcription-induced chimerism (TIC) was observed in both stickleback and zebrafish, in which chimeric transcripts are formed by alternative splicing among the original tandem genes. In stickleback, the single gene annotation contains two original α1 genes that transcribe not only their gene products, but also a chimeric transcript (atp1a1a.4-202) that bears the 5′-region of atp1a1a.4-203 and the 3′-region of atp1a1a.4-204 (Fig. 3a). In zebrafish, five annotated α1 genes are presented on the chromosome but they are not corresponding to the five original α1 genes in tandem (Fig. 3b). Instead, two of the annotated α1 genes were representing the TIC transcript among the original genes while two original genes were hidden as transcripts. In brief, zebrafish possesses five α1 genes that transcribe at least seven transcripts at the tandem α1 region (Fig. 3b).

### NKA isoform expression in the osmoregulatory organs of eel and medaka following seawater transfer

Expressions of NKA α-subunits by RNA-seq are shown in Table 3. The expression patterns were not parallel to those of real time PCR and we observed biased expression in medaka RNA-seq and many isoforms were missed in eels. We therefore mainly focused on the results obtained using real time PCR.

**Table 3:** Gene expression of various NKA α-subunits quantified by RNA-seq in the gill and intestine of Japanese medaka and eel. *RBBH* Reciprocal BLAST Best Hits

Medaka Ensembl accession no.				RNA-seq (reads per million)						Eel			RNA-seq-RBBH genes		
	Annotation	Identity		Gill (*N* = 3)			Intestine (*N* = 5)			Identity		Gill (*N* = 3)	Posterior Intestine (*N* = 5)		
			Salinity	Mean	±	SD	Mean	±	SD		Salinity		Mean	±	SD
ENSORLG	atp1a1a.4	α1a	FW	13973	±	11507	41	±	29	No entry	FW	nil	nil		
00000018557	(2 of 2)														
			50 % SW 7d	18306	±	1414	61	±	15		SW 7d	nil	nil		
ENSORLG	atp1a1a.4	α1b	FW	44604	±	23376	56974	±	11754	No entry	FW	nil	nil		
00000002122	(1 of 2)														
			50 % SW 7d	85481	±	14483	52584	±	2749		SW 7d	nil	nil		
ENSORLG	atp1a1b	α1c	FW	4948	±	948	941	±	117	α1c	FW	nil	2094	±	483
00000002047															
			50 % SW 7d	5034	±	437	952	±	152		SW 7d	nil	1599	±	695
ENSORLG	atp1a2a	α2	FW	605	±	313	2	±	1	No entry	FW	nil	nil		
00000002639															
			50 % SW 7d	348	±	70	5	±	10		SW 7d	nil	nil		
ENSORLG	atp1a3a	α3a	FW	91	±	46	20	±	8	α3	FW	nil	63	±	55
00000007036															
			50 % SW 7d	92	±	14	12	±	13		SW 7d	nil	40	±	24
ENSORLG	atp1a3b	α3b	FW	14354	±	571	318	±	99	No entry	FW	nil	nil		
00000013191															
			50 % SW 7d	21286	±	2246	241	±	70		SW 7d	nil	nil		

**Fig. 4:**
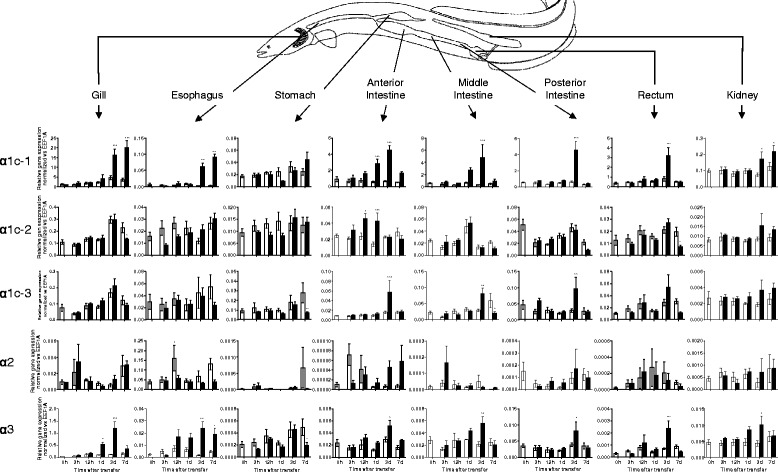
Relative gene expression of NKA isoforms in gill, esophagus, stomach, intestine (anterior, middle, and posterior), rectum, and kidney of Japanese eel quantified by real time PCR (*N* = 5). Gene expression was normalized against that of *eef1a* in each tissue. Statistical significances between each time-controlled FW-FW and FW-SW transfer groups were indicated by * (*p* < 0.05), ** (*p* < 0.01), and *** (*p* < 0.001) after tested by two way ANOVA, Tukey’s test

Using real time PCR, we measured eel NKA α-subunit expression in various tissues during a time-course after SW transfer, as shown in Fig. 4. The time-course results not only showed the long-term changes, but also revealed the transient changes during the course of SW acclimation. We observed that α1c-1 responded most significantly to SW transfer, while the expression of α1c-2 and α1c-3 were less sensitive to salinity. The α2 isoform expression was low in most tissues examined except esophagus, but again no salinity effect was observed in this isoform. High expression of α3 isoform was found in the gill and this isoform responded to salinity change in a similar pattern to those of α1c-1.

Generally, significant increases in α-subunit expression during SW transfer were observed in either of two patterns: 1) biphasic transient increase and then a decrease or 2) gradual increase and sustained elevation in acclimated fish. Transient increase in α-subunits expression was observed in α1c-1, α1c-3, and α3 in anterior, middle, posterior intestine. In addition, such pattern was also observed in α1c-1 in rectum, and α3 in gill, rectum, and kidney. In most cases of biphasic response, the transient elevation peaked at SW 3d except α1c-2 that peaked at SW 12 h to SW 1d in anterior intestine. Although we observed some late down-regulation in α1c-2 in the gill and rectum, α1c-3 in the stomach and middle intestine, and α3 in stomach, we considered the changes lacking in consistency as the expression of α-subunit isoforms in FW to FW control transfer also fluctuated in those cases. On the other hand, the stable late increase shape was observed in α1c-1 in the gill, esophagus, and kidney and α3 in the esophagus.

**Fig. 5:**
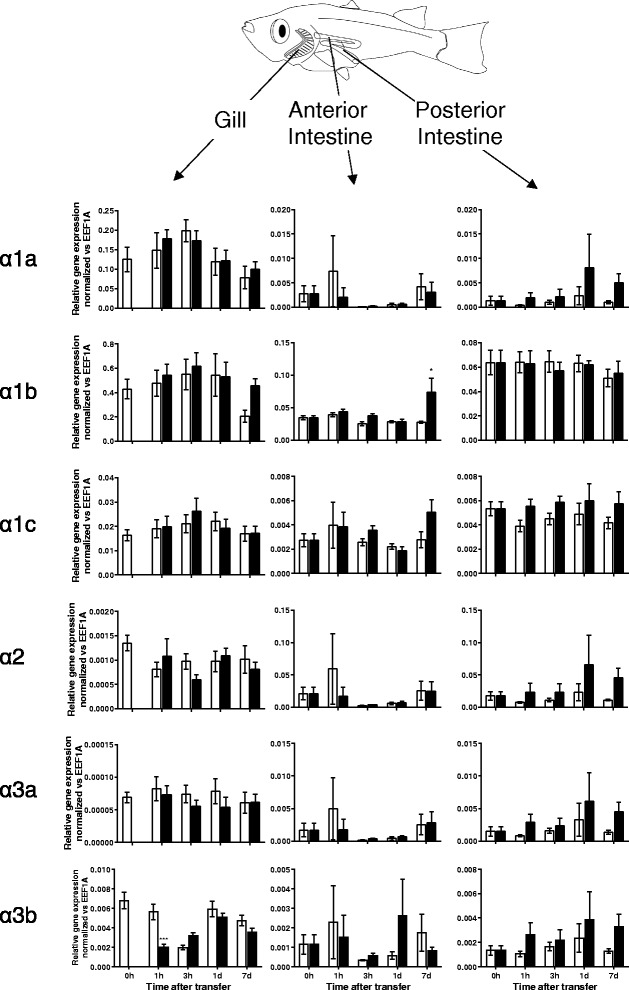
Relative gene expression of NKA α-subunit isoforms in gill and intestine (anterior and posterior) of Japanese medaka quantified by real time PCR (*N* = 6). Gene expression was normalized against that of *eef1a* in each tissue. Statistical significances between FW and SW groups within each time point were indicated by * (*p* < 0.05), ** (*p* < 0.01), and *** (*p* < 0.001) after tested by two way ANOVA, Tukey’s test

In medaka, α1b is the major expression form and it was significantly upregulated in SW7d anterior intestine but not in posterior intestine (Fig. 5). No statistical significant increase of α1b was observed in the gill but the expression was highest among all tissues examined. The expression of α2 was high in both anterior and posterior intestine but no significant changes were observed. A transient decrease in α3b expression was observed in the gill following SW transfer. Overall salinity transfer had no significant effects on the expression of other NKA isoforms in the tissues examined.

### Localization of NKA proteins and mRNAs by immunohistochemistry and in situ hybridization in Japanese eel

**Fig. 6:**
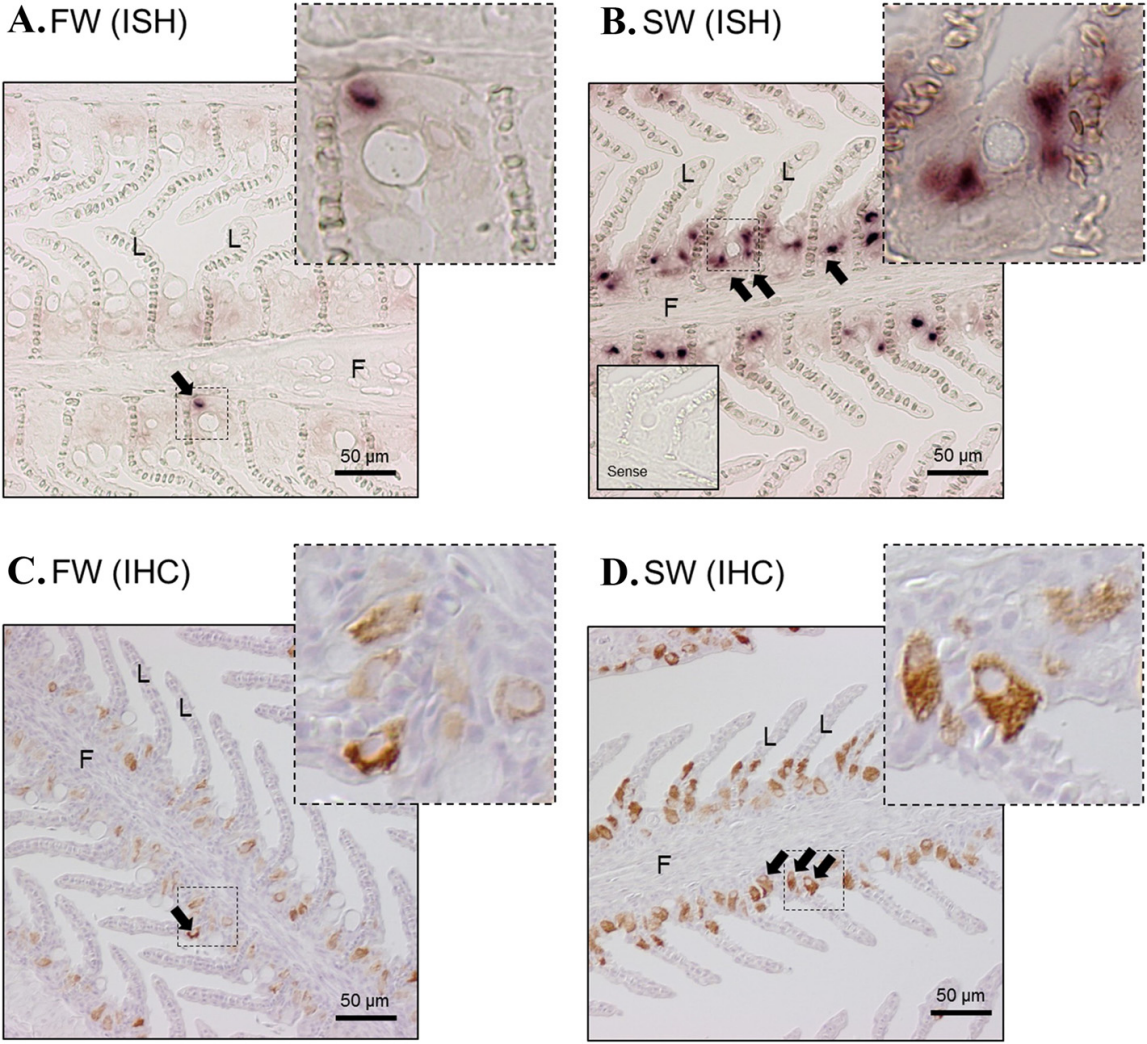
In situ hybridization of NKA α1c-1 (**a**, **b**) and immunohistochemistry of NKA protein (**c**, **d**) in the gill of eels acclimated to FW and 7 day SW. Positive signals (*arrows*) were localized at the ionocytes at the inter-lamellar epithelium. Strong and dense signals are present in SW-acclimated tissues. From immunohistochemistry, the NKA protein signals were observed at the basolateral side of the ionocytes. Top right photos show the structure of the dotted square region in high resolution. Enclosed photos show the negative control of the hybridization incubated with sense probes. F = filament; L = lamellae

**Fig. 7:**
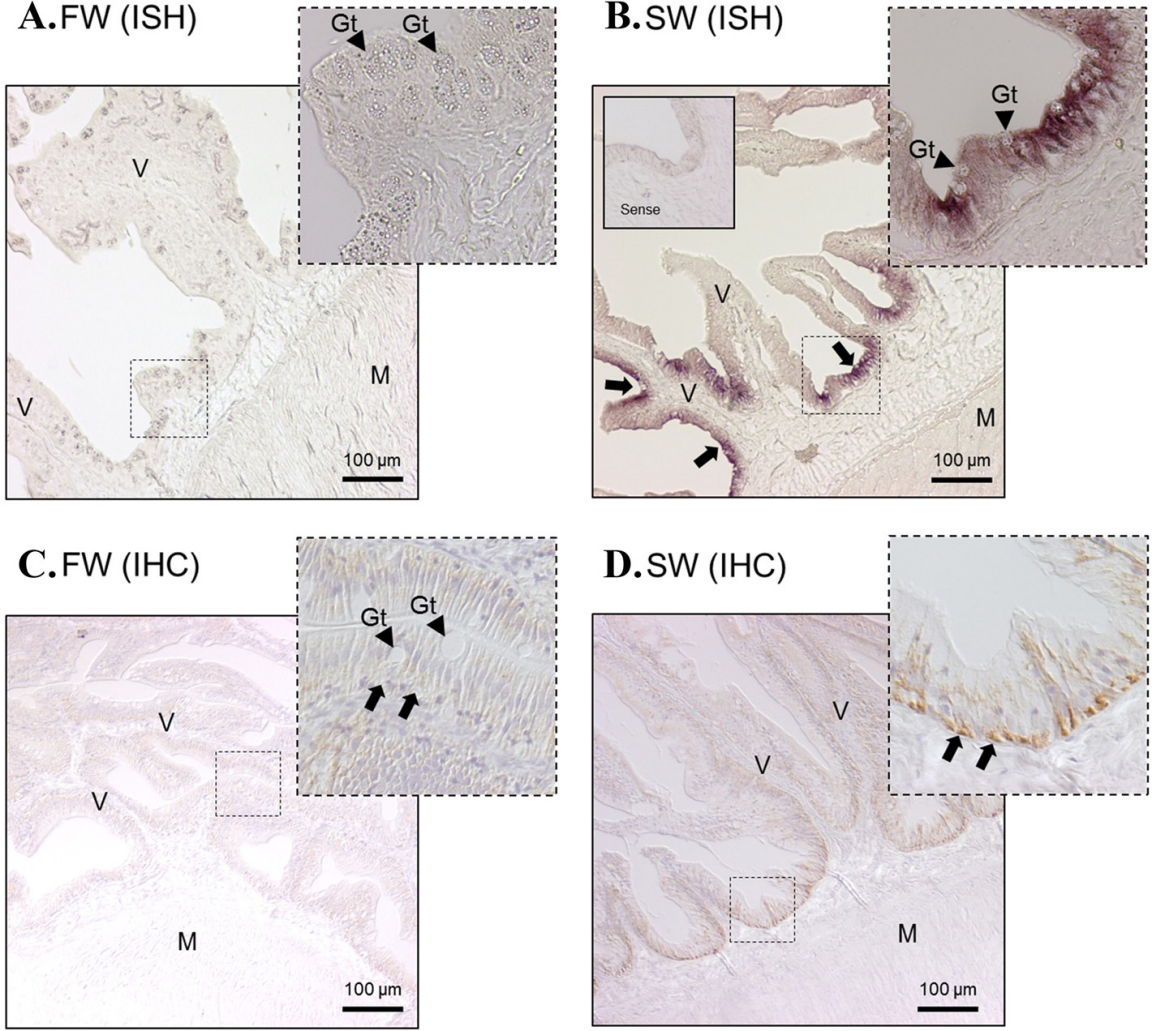
In situ hybridization of NKA α1c-1 (**a**, **b**) and immunohistochemistry of NKA protein (**c**, **d**) in the anterior intestine of eels acclimated to FW and 7 day SW. Positive mRNA signals (arrows) were localized at the mucosal epithelium. Strong and dense signals are present in SW-acclimated tissues. From immunohistochemistry, the NKA protein signals were observed at the basolateral side of the epithelium. Top right photos show the structure of the dotted square region in high resolution. Enclosed photos show the negative control of the hybridization incubated with sense probes. Arrow heads indicate the position of goblet cells (Gt). V = villus; M = muscle

**Fig. 8:**
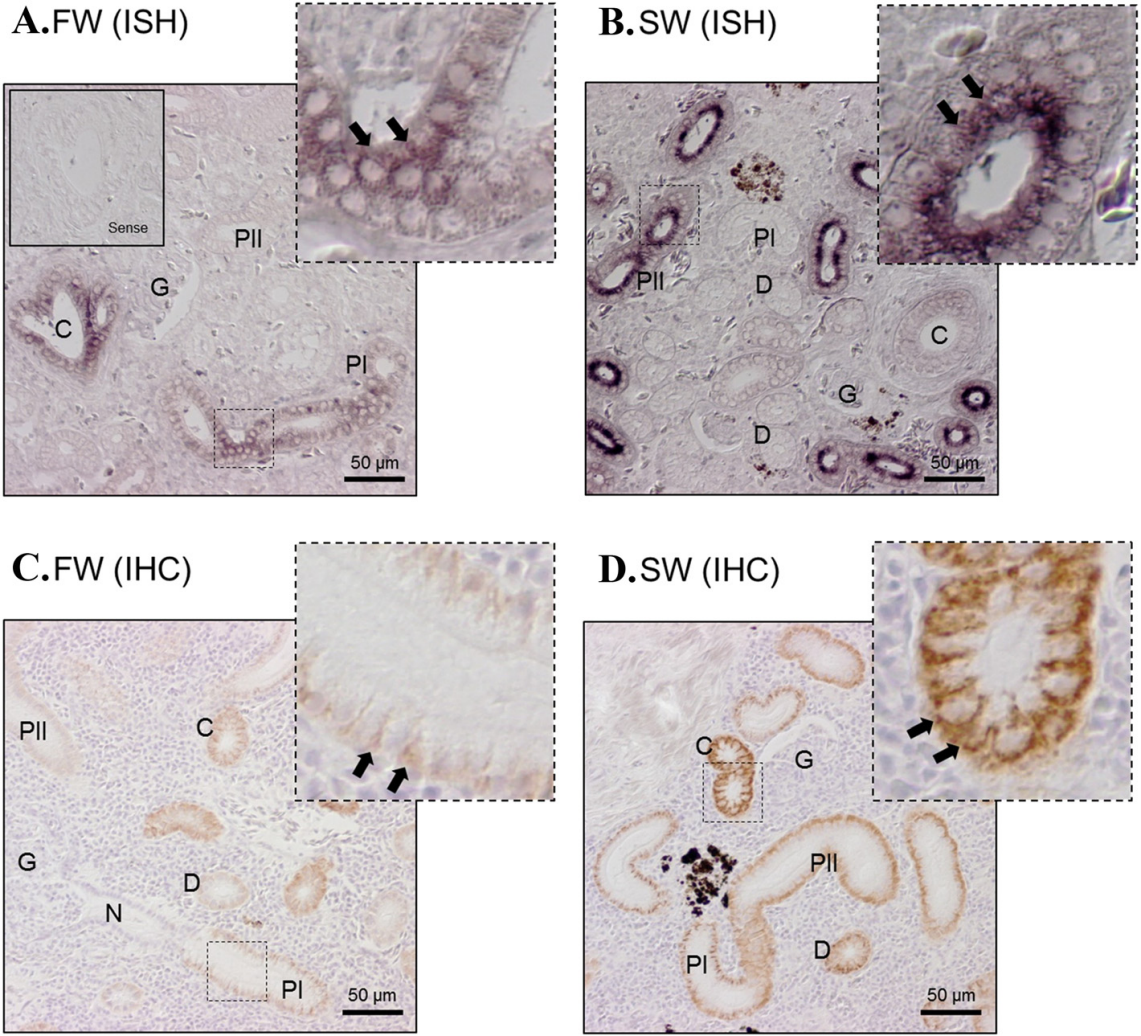
In situ hybridization of NKA α1c-1 (**a**, **b**) and immunohistochemistry of NKA protein (**c**, **d**) in the kidney of eels acclimated to FW and 7 day SW. Positive mRNA signals (arrows) were localized at the proximal tubules (both FW and SW) and collecting tubules (FW > SW). From immunohistochemistry, NKA protein signals were observed at the basolateral side of proximal tubules < distal tubules < collecting tubules. Stronger immunohistochemistry signals was generally found in SW eel compared to FW eel. Top right photos show the structure of the square region in high resolution. Enclosed photos show the negative control of the hybridization incubated with sense probes. G = glomerulus; N = neck; PI = first segment of proximal tubules; PII = second segment of proximal tubules; D = distal tubules; C = collecting tubules

Although we designed the RNA probes using the partial coding sequence and 3′-UTR to increase specificity, the probes could have hybridized to other isoforms, posing difficulties to distinguishing among isoforms. As α1c-1 is the dominant expressed isoform in the osmoregulatory epithelium, the signal contamination from α1c-1 is so overwhelming that other isoforms cannot reliably be detected and localized. Our ISH system is therefore currently limited, and thus only the result of α1c-1 localization is shown (Figs. 6, 7 and 8). In all cases, pair-wise hybridizations using sense probes did not generate in any non-specific signals in all cases. In order to compare the isoform expression and protein levels, we performed IHC of NKA protein in parallel with ISH. The antibody for NKA protein used in the present study is a well-characterized monoclonal antibody (a5, DSHB Hybridoma Bank) that has robust cross-reactivity with all tested NKA α-subunits from insects to mammals.

In the gill epithelium, the NKA protein and α1c-1 signals were located in the ionocytes at the base of the lamellar epithelium (Fig. 6), and SW transfer clearly increased the signal strength of both NKA protein and α1c-1 transcript (Fig. 6b, d). These observation consistent with the results of real time PCR. The NKA protein and α1c-1 signals were not concentrated in pavement cells or in other parts of the gill. In the anterior intestine where the α1c-1 isoform is dominantly expressed, α1c-1 signal was located at the mucosa and NKA protein was located at the basolateral membrane of the epithelium (Fig. 7). Increased intensities in both IHC and ISH were observed in SW 7d compared to FW (Fig. 7b, d), which matched with the real time PCR data. No substantial NKA signals were observed in the muscle layer and serosa.

In the kidney, the NKA protein and α1c-1 transcript signals were observed in the proximal tubules with obvious brush border (Fig. 8). From IHC, NKA protein signals were observed at the basolateral membrane of proximal tubules, distal tubules (D), collecting tubules (C) in order of increasing signal. Stronger immunohistochemistry signals were generally detected in SW eel compared with FW eel. The first segment of proximal tubules (PI) with basolateral nucleus and columnar cells expressed weak α1c-1 transcript while the second segment of proximal tubules (PII) with central nucleus expressed relatively stronger α1c-1 signal, similar to the pattern observed in IHC. However, the distal tubules with cuboidal cells and collecting tubules surrounded by connective tissues expressed low α1c-1 signal in SW compared with FW, which contrasts with the results of IHC, which showed higher NKA protein contents in distal and collecting tubules. Glomeruli were negative of signal in both IHC and ISH.

## Discussion

### Sequence characteristics of NKA α-subunit isoforms and expression patterns after seawater transfer

In this first comprehensive study of eel NKA α-subunit isoforms, we revealed some fundamental differences in isoform compositions and expressions between eel and other species. The most striking finding is that eels apparently do not possess the α1a and α1b isoforms as salmonids and other species do, but instead α1c was replicated into three isoforms. The uniqueness of eels in this regard led us to re-examine the possible evolutionary scenarios of teleost NKA α-subunits. We combined the gene expression and phylogenetic results to address the evolutionary selection of NKA α-subunits in teleosts in relation to osmoregulation and to resolve a confusing nomenclature.

Among the three eel α1c isoforms, the α1c-1 isoform responded most intensely to SW acclimation in various osmoregulatory organs (Fig. 4). The biphasic expression responses in the intestine indicated that the transitional change of transporter properties in the epithelium was maximum at SW 3d. This also mirrored the plasma osmolality results where peak osmolality was observed at SW 3d and the osmolality returned to pre-transfer value after SW 7d [25]. This transient change suggests that transporter composition in eel intestinal epithelium was reorganized its during SW 1d to SW 3d, and that a new functional epithelium specialized in water absorption was formed after SW 7d. In the gill, esophagus, and kidney, however, the α1c-1 upregulation was continuous instead of transient, which indicated a sustained necessity for active transport in these tissues. The NKA in the gill ionocytes is well-known to be the major driving force for ion excretion via basolateral Na-K-Cl cotransporter (NKCC1) and apical cystic fibrosis transmembrane conductance regulator (CFTR) [26]. Eel esophagus is an important osmoregulatory organ to desalinate SW rapidly to approximately 50 % [27, 28], but the molecular mechanism of desalination remains poorly understood. We found that the α1c-1 and α3 were upregulated in the SW esophagus but their roles in desalination in SW eel was not clear. Expression of NKA α-subunits in eel stomach was not affected by salinity changes, indicating the organ is not related to desalination or water absorption but major for digestion.

We also observed a higher expression of α1c-1 transcript in the SW eel kidney although previous studies have reported a higher NKA protein and expression level in FW [29, 30]. In a previous IHC study in eels [30], an antiserum raised against the conserved region of NKA was used and we examined the antigen peptide epitope [31], which possess a sequence that is identical in all eel isoforms identified so far, thus should give a robust cross-reactivity to NKA isoform in eel tissues. From our IHC results, higher NKA protein levels were found among the kidney tubules in SW eels, which coincided with the results obtained in real time PCR (Fig. 4). The NKA activity in the kidney of European eel in SW was 2.5-fold higher than in FW [32], which coincides with the higher α1c-1 expression in SW eels. NKA activities in renal tubules are important in both FW and SW [30]. A high glomerular filtration rate is necessary in FW to remove excess water, thus high NKA activity to drive Na and Cl reabsorption rates is necessary. In SW, glomerular intermittency limits the glomerular filtration and the active secretion of Mg^2+^ and SO_4_^2-^ is prominent via anion-exchangers *slc26a1* and *slc26a6* at the proximal tubules [33]. High Na^+^/H^+^ antiporter 3 (NHE3) protein was found on the apical membrane of PII tubules in SW eel, suggesting a role in the reabsorption of Na^+^ from glomerular filtrate to generate an osmotic gradient to drive water reabsorption [34]. High NKA protein levels in the distal and collecting tubules in SW eel suggest high reabsorption of Na^+^ to reabsorb water for conservation, leaving high Mg^2+^ and SO_4_^2-^ concentration in the urine. However, from our ISH results, the α1c-1 expression in the distal and collecting tubules was low or undetectable, which did not match with the high protein levels shown by IHC (Fig. 8). This result indicated that a SW-specific regulation of NKA α1c isoforms is present in eel distal and collecting tubules. The discrepancy between ISH and IHC results was not due to alternative transcripts of α1c-1 or isoform switching, since an alternative cRNA probe against the conserved region of all NKA isoforms, and an alternative NKA antiserum resulted in the same ISH and IHC patterns respectively. Further investigations are necessary to elucidate the possible reasons for this discrepancy between protein and mRNA distribution and turnover rates in the distal and collecting tubules of SW eel kidney.

The α2 expression was generally low in most tissues but in esophagus expression was relatively high (Fig. 4). The α2 could be located on the skeletal muscle as the esophagus is one of the internal organs that possess striated muscle, and concurrently α2 was expressed mainly in red and white muscles in rainbow trout [4]. Mammalian α2 is also present mainly in skeletal muscles and involved in contraction [35]. However, we also observed that α2 expression is high in the intestine of medaka (Fig. 5), so it is possible that α2 is also expressed in smooth muscles in medaka or it may have other species-specific new functions. There are few literature reports on the expression changes in α2 and α3 in teleosts under salinity challenge conditions, but we have demonstrated that eel α3 expression is salinity sensitive, which followed mostly the changes of major isoform α1c-1 (Fig. 4). In stickleback, α3b expression was not sensitive to salinity acclimation [12], but α3 was upregulated in the gill of FW killifish [36] and SW tilapia [37]. Salinity specific changes in α3 is intriguing as α3 is exclusively expressed in the nervous system in mammals [38], and thus it is possible that α3 is involved in the remodeling of neurons. The α1c-2 and α1c-3 expressions are less responsive during SW acclimation, suggesting their contributions in salinity acclimation are minor (Fig. 4). Whether their expression are redundant or supportive to α1c-1 required further studies.

In medaka, the expression of NKA α-subunits showed little sensitivity to SW acclimation. Only α1b was found to be significantly upregulated in the anterior intestine after SW acclimation. Although a tendency of increase in the branchial expression of α1b between FW7d and SW7d was observed (Fig. 5), the difference was not statistical significant because of the sustained high expression levels in both control and salinity transfers at other time points. This suggests that the α1b could be stimulated by adrenergic and/or cortisol responses, where handling stress plays an important role. The expression of α3b in the gill following SW transfer downregulated transiently but rapidly resumed to the pre-transfer level after SW1d, suggesting that the branchial neurons may be degenerated during the initial SW acclimation as α3 is most abundant in the nervous system [38]. After SW1d, the α3b was resumed as newly developed neurons were replaced. Other isoforms were not salinity sensitive and our results were highly similar to those reported by others recently [9]. Although the lack of changes in expression of NKA α-subunits seems puzzling as dramatic reorganization of osmoregulatory epithelia was expected, other levels of regulatory control such as translation stimulation/inhibition and protein activity/trafficking control by regulatory proteins such as FXYD protein [39, 40] should be considered in future studies.

### Relationship between real time PCR time-course changes and transcriptome analysis

RNA-seq is not well suited to the analysis of closely similar genes, such as NKA α-subunits. Our group observed biased expression results for some isoforms with higher intrinsic expression when we analyzed the transcriptomes of eel and medaka tissues (Table 3). From our transcriptome results, many NKA α-subunit isoforms were apparently missed in the eel transcriptome and biased expression of α1b was observed in medaka. The biased expression results can probably be attributed to the short reads produced by Illumina sequencing. These short reads failed to tag the correct isoform because the reads could have identical sequences to several isoforms in the database, resulting in a biased count for a particular isoform. Given that the NKA isoforms carry important implications for osmoregulatory functions in teleost tissues, we have resolved their expression by cloning all possible α-subunit isoforms in eel and medaka. Given the differences between the RNA-seq and real time PCR results, the RNA-seq results should be interpreted conservatively, especially since teleosts possess similar isoforms from independent or 3R whole genome duplications.

Our time-course data show that transient changes in osmoregulatory transporter transcript levels matched the physiological changes in fish during the course of salinity acclimation [25]. These patterns are an important basis for our future analyses of transcriptome data since transcription factors and regulatory hormones that regulate the transporter expression are often transiently involved. There must be awareness of expression patterns, temporal schemes, tissue types, are species-specific differences. For example, in eel intestine, the α1c-1 upregulation started after SW 1d and peaked at SW 3d, thus implying that the controlling transcription factors could be upregulated shortly prior to the transporter upregulation. However, in eel gill and kidney, α1c-1 upregulation was late and continuous, suggesting that the transcription factors that regulate α1c-1 are tissue-specific. In medaka, transient downregulation of α3b in the gill following SW transfer indicated that specific transcription factors could be targeting this isoform, and the entire regulatory phase lasted only a few hours. Comparison between the expression profiles of NKA α-subunit isoforms of medaka and eel clearly showed lineage-specific selection of isoforms selected in osmoregulation.

### Evolutionary scheme of teleost NKA α-subunits from the perspective of salinity acclimation

**Fig. 9:**
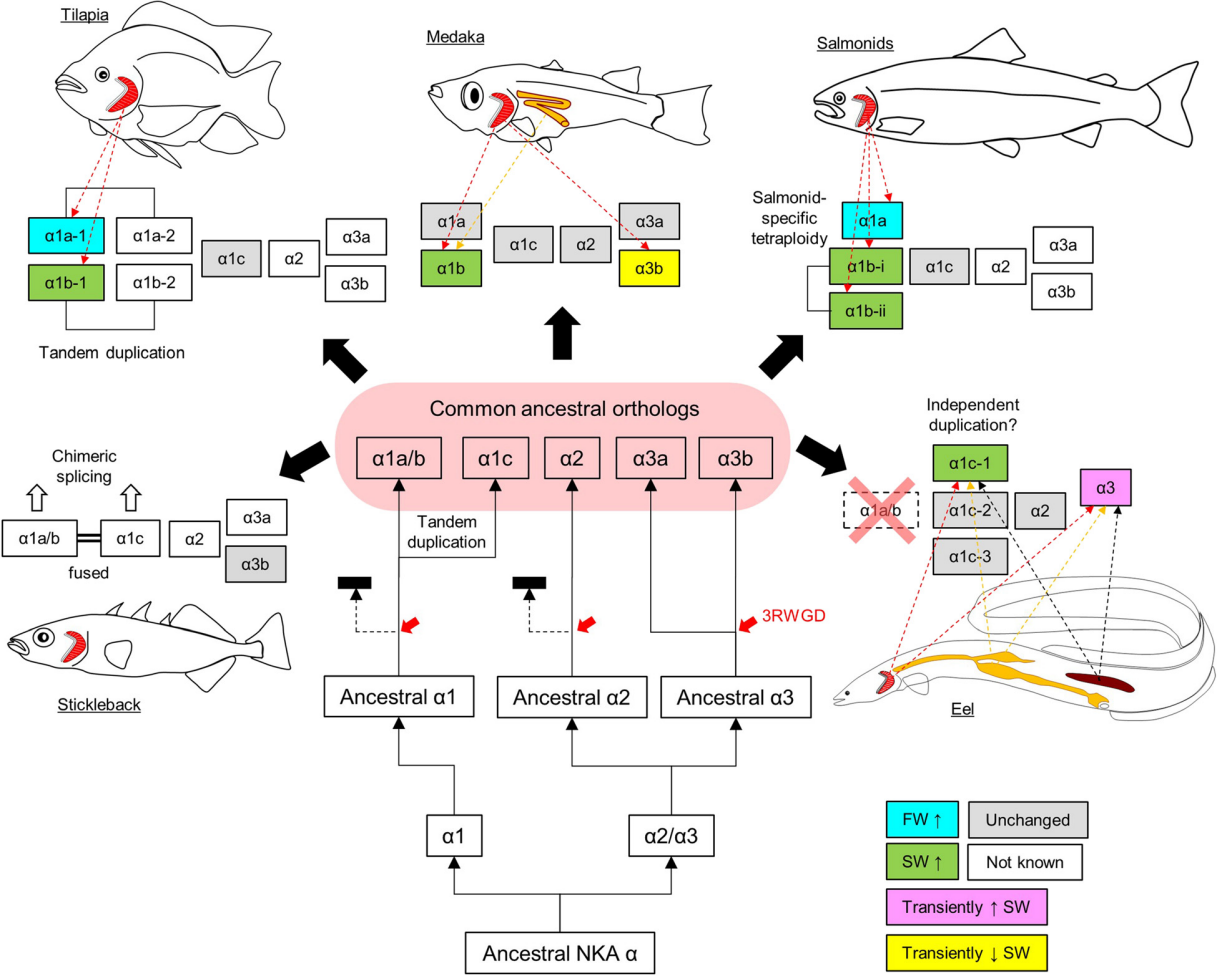
Evolutionary scheme of NKA α-subunit in teleosts with various strategies adopted by different lineages. Multiple α1 isoforms/transcripts were generated by tandem gene duplication, gene fusion, alternative splicing. The α1a and α1b among different species are non-monophyletic and were generated via different lineage-specific duplication events. The isoform composition (*pink shaded*) indicates the hypothetical condition before divergence of different teleost lineages. Known changes in gene expression in known organs (indicated by colored *dotted arrows*) during salinity transfer are shown in different colors. Red dotted arrow = gill-specific; orange dotted arrow = intestine-specific; black dotted arrow = kidney-specific. 3RWGD = teleost specific 3rd round whole genome duplication

Our results show the diverse nature of NKA α-subunits in teleosts. We propose a scenario for the adaptive radiation of these subunits, with special focus on the α1 subclades (Fig. 9). We emphasize that the α1a and α1b described here are gene isoforms or transcripts that are paralogs, which were produced by independent or tandem duplications within α1a/b subclade, but shared similar functions at the time when they were described. Since the nomenclature is complicated and confusing, we included Table 2 for the readers to match the accession numbers, genome annotation, and conventional isoform names commonly used by fish physiologists. It is important to note that the genome annotation often refers α1a/b as *atp1a1a* while α1c as *atp1a1b*, which is not surprising as the phylogenetic relationship (Fig. 1) shows two clear subclades corresponding to *atp1a1a* and *atp1a1b*. It is thus important to avoid confusing α1b with *atp1a1b*. The problematic genome annotations also confused alternative transcripts from genes and thus clarification was made on Table 2 when appropriate. We included most common fish models used in osmoregulation studies and analyzed their genomic structures, when possible, to avoid alternative transcripts in the phylogenetic analysis. The phylogenetic analysis aims to depict the diversification of NKA α-subunit isoforms and the relationship is different from a species tree that states the phylogenetic relationship among species. We included a contemporary species tree [24] of the representative species (Fig. 1) to aid readers to compare and contrast the diversification between proteins and species.

The phylogeny results suggest that the ancestral NKA α-subunit gene first duplicated into α1 and α2/α3, and the latter further duplicated and radiated into the extant α2 and α3 clades. In the α3 clade but not α1 and α2 clades, it is clear that the teleost-specific 3R whole genome duplication generate α3a and α3b, which were retained in extant teleost while the duplicated α1 and α2 clades were possibly lost. Whether the eel α3 is α3a or α3b is still questionable, as the phylogenetic analysis showed that it is grouped into α3a clade, but the synteny result suggested either clade. The existence of α3b in eel thus requires further sequencing information. The α3 subclade may have developed a supportive role to α1 as the expression patterns between α1 and α3 were similar in various species studied so far (Fig. 4) [12, 36, 37]. Note however that α2 expression is not sensitive to salinity changes, which is reasonable as α2 was specially found in muscles and may be related to muscle contraction and nerve impulse [41].

The ancestral α1 duplicated into α1a/b and α1c by tandem duplication is shown by the synteny results (Fig. 2), and this basic composition underwent different evolutionary pathways in teleosts as demonstrated by the representative species shown in Fig. 9. In salmonids, α1c was not duplicated and functioned as house-keeping NKA, as its expression is not sensitive to salinity transfers [4–6]. The α1a/b duplicated into α1a and α1b and were functionally specialized in FW and SW ionocytes respectively. Two α1b isoforms (α1b-i and α1b-ii) were known in Atlantic salmon due to the salmon-specific tetraploidy, but their functional specializations are not known. Although the switching of NKA α1a and α1b upon salinity transfer was well-demonstrated [5], the functional roles of other isoforms require further studies.

In tilapia, the NKA α1c was house-keeping as in salmon but the α1a/b experienced two tandem duplications, forming four α1 isoforms (Fig. 2). Since the phylogenetic results suggested that the duplications were independent to that of salmonid, the α1a-1, α1a-2, α1b-1, and α1b-2 isoforms were paralogous to those of salmonids. The tilapia α1a-1 and α1b-1 behaved similarly to those of salmonid in salinity acclimation [7], but no expression information is yet available for α1a-2 or α1b-2.

In medaka, NKA α1a and α1b genes are situated on different chromosomes, suggesting that the duplication from α1a/b could be independent rather than tandem as in tilapia. The resultant medaka α1a and α1b are paralogous to those of salmon and tilapia. The gene expression of medaka α1b was similar to those of salmonids and tilapia, but α1a did not downregulate in either gill or intestine following SW transfer (Fig. 5) [9]. As shown in Fig. 1, the combination of α1a and α1b of salmon, tilapia, and medaka generated a confusing paralogous relationship within the α1a/b subclade, thus we should consider a new nomenclature system based on phylogenetic relationships rather than functional characteristics in salinity acclimation.

The selection of α-subunits in osmoregulation appear surprisingly flexible when we analyzed the NKA α-subunits in eel. The eel α1a/b paralog was probably deleted in early anguilliform, and α1c was selected to perform the lost functions. Such selection pressure may have promoted duplications and divergence in α1c subclade into several isoforms, and α1c-1 was selected as the major isoform upregulated in SW. This is the first demonstration that besides α1a and α1b switching, alternative α-subunits were selected as the major isoform for osmoregulation. From the species tree in Fig. 1, the eel family spun off from the main stream early in the evolution of teleost, thus they may have accumulated more substitutions and shuffling during the evolution. Therefore, we should consider that more exceptional cases may arise when more sequencing and expression data are available.

The flexibility of α1 isoform selection was further shown in the case of stickleback. Unlike eel, the stickleback family is evolutionary recent (Fig. 1), which may implicate some alternative selections of isoforms for osmoregulation. The ancestral gene of α1a/b was not duplicated or the duplicated copy was lost. The side-by-side α1a/b and α1c isoforms were considered as a single gene by genome annotation (Fig. 3a) with alternative splicing generating a novel chimeric α-subunit (atp1a1a.4-202) along with two parent gene products (atp1a1a.4-203 and atp1a1a.4-203). The transcript atp1a1a.4-201 is slightly different from atp1a1a.4-203 at the 5′-region, thus we did not consider the phylogenetic difference between this two transcripts, but physiological studies in the future should consider the difference in expression regulation of these chimeric and alternative transcripts. Expression of transcript atp1a1a.4-201 and atp1a1a.4-204 were specifically examined in stickleback in relation to sexual maturation, and in the intestine but not kidney, mature female possessed significantly higher expressions of both isoforms than immature male [42]. The primers used were specific to the 5′-regions of both transcript atp1a1a.4-201 and atp1a1a.4-204, thus did not detect the expression of the other two isoforms. On the other hand, salinity effects on the expression of these alternative transcripts, along with other novel chimeric transcripts, have not been reported yet, and thus further studies are required for their roles in osmoregulation. In a previous study, the expression pattern of stickleback *atp1a1* was analyzed by microarray, but the hybridization-based method probably could not have distinguished the isoforms [43]. *atp1a1* was also considered to be a single gene, as suggested by the stickleback genome, and thus the isoforms within the same locus were not examined independently [44]. Some proposed that *atp1a3* is the major NKA isoform and reported few changes in expression among salinities [12].

The TIC phenomenon is not unique to stickleback as we observed a similar event in zebrafish (Fig. 3b). We closely examined the genomic region on zebrafish chromosome 1, which carries the α1 genes, and found five α1 gene structures located tandemly in the same orientation. However, the five annotated genes reported in this region transcribe at least seven known transcripts, where two alternatively transcribe across the five α1 genes; i.e. five genes but seven transcripts (Fig. 3b; Table 2). Unlike in stickleback, the alternative transcripts among five α1 genes were interpreted as independent genes in zebrafish, while the original genes were hidden as transcripts. We should thus be cautious on the genome annotations and also consider the complexity of isoform composition in each species. TIC is a common phenomenon in eukaryotes and at least 2–5 % of the genes in the human genome were involved in these events [45, 46]. TIC increases the protein complexity and the chimeric gene could be duplicated to another locus to form new genes. We observed TIC among the tandem duplicates of parent α1 genes in both zebrafish and stickleback, indicating that such phenomenon could be also possible in other species. However, there are so far no generally accepted rules to name the TIC products so that the distinction between genes and transcripts is ambiguous, thus fish physiologists should be cautious on describing the identity of NKA α1 isoforms in their model organisms.

The switching of NKA α1a and α1b isoforms between FW and SW, respectively, is not universal in teleosts and our results showed that the selections of α1 isoforms were flexible. The duplicated α1 isoforms were similar in terms of structure and functions, thus the duplicated isoforms could have equal potential to play a central role in salinity acclimation. The selection of one isoform to be dominantly expressed isoform followed one or more duplication of an ancestral gene, was lineage-dependent. In functional domain perspective, the conserved domain for α1a and α1b could be due to strong functional constrains [8]. Although a tendency for increasing number and complexity of α1 transcripts in different lineages through various duplications and TIC splicing was observed, the large number of isoforms does not necessarily relate to euryhalinity since zebrafish possess several α1 genes and transcripts and yet it is a stenohaline FW species. Increases in isoform number of the enzymes with the same basic function increases the robustness of the biological system by enzyme proportion control to compensate each other under different environmental stresses [47]. However, the putative selection pressure for a high NKA α1 isoform complexity was not yet identified.

## Conclusion

NKA α-subunits are highly diversified in teleost fishes. It is likely that the duplicated genes have undergone parallel evolution under similar functional constraints, and now perform the same physiological function in the different fish lineages. Various mechanisms, such as independent tandem duplications and alternative splicing among different isoforms, were adopted to increase the structural and functional complexity of NKA α-subunits. Combining the results of gene expression and phylogenetic relationship, we demonstrated the diverse nature of NKA α-subunits in various teleost lineages and a possible evolutionary scheme relative to isoform selection for osmoregulation. The nomenclature of NKA α-subunits should be reviewed further to establish a common consensus among fish researchers. Contrasting results from transcriptome and quantitative PCR studies suggest that the former method is not reliable for NKA α-subunit and thus these closely-related genes should be separately studied.

## Abbreviations

3RWGD, the third round whole genome duplication; CFTR, cystic fibrosis transmembrane conductance regulator; differential expressed genes; FW, freshwater; IHC, immunohistochemistry; ISH, In situ hybridization; NGS, next-generation sequencing; NKA, Na^+^/K^+^-ATPase; NKCC1, Na-K-Cl cotransporter; ORF, open reading frame; RBBH, reciprocal BLAST best hit; SW, seawater; TIC, transcription-induced chimerism; UTR, untranslated region
